# Consensus recommendations for an integrated diagnostic approach to peripheral nerve sheath tumors arising in the setting of Neurofibromatosis Type 1

**DOI:** 10.1093/neuonc/noae235

**Published:** 2024-11-06

**Authors:** Calixto-Hope G Lucas, Andrea M Gross, Carlos G Romo, Carina A Dehner, Alexander J Lazar, Markku Miettinen, Melike Pekmezci, Martha Quezado, Fausto J Rodriguez, Anat Stemmer-Rachamimov, David Viskochil, Arie Perry, the Symposium on Atypical Neurofibroma: State of the Science Members, Shivani Ahlawat, Srivandana Akshintala, Kimberly Amrami, Annette Bakker, Allan Belzberg, Jaishri O Blakeley, Miriam Bredella, Prashant Chittiboina, D Wade Clapp, Heike Daldrup-Link, Thomas De Raedt, Carina Dehner, Eva Dombi, Garrett Draper, Laura Fayad, Rosalie Ferner, Michael J Fisher, David H Gutmann, Andrea M Gross, Kristina Hawk, Angela Hirbe, Fabian Johnston, Aerang Kim, Bruce R Korf, David Largaespada, Alexander Lazar, Lu Le, Eric Legius, Adam S Levin, Calixto-Hope G Lucas, Ina Ly, Markku Miettinen, David Miller, Carol Morris, Mark Murphey, Luis Parada, Melike Pekmezci, Arie Perry, Christine Pratilas, Martha Quezado, Marcus Ratley, Nancy Ratner, Steven D Rhodes, Inka Ristow, Fausto Rodriguez, Carlos G Romo, Eduard Serra Arenas, Steven Sheard, John Shern, Benjamin Siegel, Anat Stemmer-Rachamimov, R Taylor Sundby, Jeffrey Szymanski, Harish N Vasudevan, David Viskochil, Brian D Weiss, Lennart Well, Brigitte C Widemann.

**Affiliations:** Department of Oncology, Johns Hopkins University School of Medicine, Baltimore, Maryland, USA; Department of Neurosurgery, Johns Hopkins University School of Medicine, Baltimore, Maryland, USA; Department of Pathology, Johns Hopkins University School of Medicine, Baltimore, Maryland, USA; Pediatric Oncology Branch, National Cancer Institute, National Institutes of Health, Bethesda, Maryland, USA; Department of Medicine, Johns Hopkins University School of Medicine, Baltimore, Maryland, USA; Department of Neurology, Johns Hopkins University School of Medicine, Baltimore, Maryland, USA; Department of Oncology, Johns Hopkins University School of Medicine, Baltimore, Maryland, USA; Department of Pathology, Indiana University, Indianapolis, Indiana, USA; Departments of Pathology and Genomic Medicine, The University of Texas MD Anderson Cancer Center, Houston, Texas, USA; Laboratory of Pathology, National Cancer Institute, National Institutes of Health, Bethesda, Maryland, USA; Department of Ophthalmology, University of California San Francisco, San Francisco, California, USA; Department of Pathology, University of California San Francisco, San Francisco, California, USA; Laboratory of Pathology, National Cancer Institute, National Institutes of Health, Bethesda, Maryland, USA; Department of Pathology, University of California Los Angeles, Los Angeles, California, USA; Department of Pathology, Massachusetts General Hospital, Boston, Massachusetts, USA; Department of Pediatrics, University of Utah, Salt Lake City, Utah, USA; Department of Neurological Surgery, University of California San Francisco, San Francisco, California, USA; Department of Pathology, University of California San Francisco, San Francisco, California, USA

**Keywords:** consensus, guidelines, molecular neuropathology, nerve sheath tumor, neurofibromatosis type 1

## Abstract

Consensus recommendations published in 2017 histologically defining atypical neurofibromatous neoplasm of uncertain biologic potential (ANNUBP) and malignant peripheral nerve sheath tumor (MPNST) were codified in the 2021 WHO Classification of Tumors of the Central Nervous System and the 2022 WHO Classification of Tumors of Soft Tissue and Bone. However, given the shift in diagnostic pathology toward the use of integrated histopathologic and genomic approaches, the incorporation of additional molecular strata in the classification of Neurofibromatosis Type 1 (NF1)-associated peripheral nerve sheath tumors should be formalized to aid in accurate diagnosis and early identification of malignant transformation and enable appropriate intervention for affected patients. To this end, we assembled a multi-institutional expert pathology working group as part of a “Symposium on Atypical Neurofibroma: State of the Science.” Herein, we provide a suggested framework for adequate interventional radiology and surgical sampling and recommend molecular profiling for clinically or radiologically worrisome noncutaneous lesions in patients with NF1 to identify diagnostically-relevant molecular features, including *CDKN2A/B* inactivation for ANNUBP, as well as *SUZ12*, *EED*, or *TP53* inactivating mutations, or significant aneuploidy for MPNST. We also propose renaming “low-grade MPNST” to “ANNUBP with increased proliferation” to avoid the use of the “malignant” term in this group of tumors with persistent unknown biologic potential. This refined integrated diagnostic approach for NF1-associated peripheral nerve sheath tumors should continue to evolve in concert with our understanding of these neoplasms.

## Background

Peripheral nerve sheath tumors arising in the setting of the Neurofibromatosis Type 1 (NF1) cancer predisposition syndrome constitute a histologically and molecularly diverse collection of tumors, where an accurate classification through minimally invasive sampling (ie, large bore core biopsy) is often sought to guide clinical decision making.^1,2^ The majority of cutaneous and visceral tumors are histologically best classified as neurofibromas. However, some noncutaneous tumors (ie, associated with large nerves or nerve plexi) may harbor worrisome morphologic features warranting consideration of more biologically aggressive entities. In this regard, high-grade malignant peripheral nerve sheath tumors (MPNSTs) demonstrate brisk mitotic activity and areas of tissue necrosis. Another subset of nerve sheath tumors may demonstrate more subtle worrisome features and such tumors lacking conventional “high-grade” MPNST-defining histology have been classified as either an atypical neurofibromatous neoplasm of uncertain biologic potential (ANNUBP) or a “low-grade” MPNST. Atypical neurofibromatous neoplasm of uncertain biologic potential and MPNST often arise from a preexisting lower-grade precursor lesion, and intratumoral heterogeneity may further complicate efforts to achieve accurate classification that properly guides clinical management.^3,4^

A consensus conference held in 2016, and subsequent consensus recommendation published in 2017, defined ANNUBP as a peripheral nerve sheath tumor exhibiting a minimum of 2 of the following features: (a) cytologic atypia, (b) loss of neurofibroma architecture, (c) hypercellularity, or (d) a mitotic count over 1/50 high-power fields (HPFs) but less than 3/10 HPF (Table 1).^5,6^ In contrast, low-grade MPNST referred to non-necrotic tumors with morphologic features of ANNUBP but a mitotic count of 3–9/10 HPF (Table 1). Most studies highlight highly overlapping genetic features and clinical behavior between ANNUBP and low-grade MPNST, although large series are lacking for these relatively rare subtypes.^7–9^ These consensus histologic criteria have since been codified in the 2021 WHO Classification of Tumors of the Central Nervous System and the 2022 WHO Classification of Tumors of Soft Tissue and Bone.^10,11^

**Table 1.** 2017 Consensus Histologic Criteria

ANNUBP	Neurofibromatous neoplasm with at least 2 of 4 features: cytologic atypia, loss of neurofibroma architecture, hypercellularity, and mitotic index > 1/50 HPFs and <3/10 HPFs
MPNST, low-grade	Features of ANNUBP, but with mitotic index of 3–9/10 HPFs and no necrosis
MPNST, high-grade	MPNST with at least 10 mf/10HPFs or 3–9 mf/10 HPFs combined with necrosis

ANNUBP = atypical neurofibromatous neoplasm of uncertain biologic potential; HPFs = high-power fields; MPNST = malignant peripheral nerve sheath tumors.

Along with the introduction of the above morphology-based classification scheme and the use of molecular data, contemporaneous multi-omic studies have examined molecular drivers across the spectrum of NF1-associated nerve sheath tumors. While the spectrum of peripheral nerve sheath tumors arising in the setting of NF1 typically exhibit biallelic *NF1* gene inactivation with loss of neurofibromin protein expression, additional molecular events have been described in the subset of tumors with worrisome histologic and clinical features.^12–17^ Though the natural history of NF1-associated neurofibroma and high-grade MPNST are well-defined, there is far less clarity regarding the clinical natural history of ANNUBP or low-grade MPNST, and it is not known if such lesions truly require oncologic surgical resections or radiation as is often pursued for high-grade MPNST. In light of this clinical need and the extensive evidence of specific molecular profiles across the spectrum of NF1-associated nerve sheath tumors, there is a strong need to incorporate these molecular alterations into an integrated diagnostic scheme to maximize clarity and accuracy in diagnosis. To this end, we assembled a multi-institutional expert pathology working group as part of a “Symposium on Atypical Neurofibroma: State of the Science” held April 11–12, 2024, at the National Institutes of Health in Bethesda, Maryland. This symposium was cosponsored by the National Cancer Institute Pediatric Oncology Branch and the Neurofibromatosis Therapeutics Acceleration Program and brought together international experts to form 4 working groups: pathology, clinical/surgical, imaging, and preclinical/translational. All participants and their affiliations are listed in Supplementary Table 1. Here, we provide the consensus rationale from the symposium for an integrated diagnostic approach for ANNUBP and MPNST.

### Copy Number Alterations

The majority of neurofibromas profiled to date demonstrate near-diploid genomes. In addition, homozygous deletion of the *CDKN2A/B* cell cycle regulator is a notable frequent and likely early initiating copy number alteration event seen in ANNUBP and MPNST.^2,18–29^ Across studies, *CDKN2A/B* deletion is typically the only other genomic alteration noted aside from the loss of chromosome 17q and *NF1* gene expression in ANNUBP. Atypical neurofibromatous neoplasm of uncertain biologic potential otherwise demonstrates balanced genomes with no other recurrent copy number alterations. Spatial profiling has confirmed *CDKN2A/B* copy number loss in neurofibromas with histologic transition to ANNUBP.^19^ Homozygous *Cdkn2a* inactivation was also shown to drive the malignant transformation of *Nf1*^−/−^ Schwann cells in genetically engineered mice, further implicating this gene in the progression of NF1-associated peripheral nerve sheath tumors.^28^ Interestingly, heterozygous inactivation of *Cdkn2a* in mice was sufficient to lead to tumor formation with complete loss of p16 protein, and heterozygous deletion of *CDKN2A/B* has also been reported in various clinical cases of ANNUBP.^20,25^ Moreover, a subset of MPNST harbor polyploid or highly aneuploid genomes, including gains and losses across multiple chromosomes.^23,25,27,29–33^ In longitudinal sampling studies, chromosomal gains and losses were only identified in MPNST and not in precursor lesions.^32^

### Short Structural Variants

Notably, a large subset of MPNST harbor inactivating *SUZ12* or *EED* mutations, subunits of the PRC2 complex.^25,29,31,34–36^ Enrichment for these alterations also aligns with H3K27me3 loss in the majority of MPNST. In addition, other events such as *TP53* mutation have also been noted in biologically aggressive peripheral nerve sheath tumors.^30–32,37^ While infrequent, *TP53*-altered MPNST have worse clinical outcomes relative to *TP53*-wildtype cases.^38,39^ In zebrafish, *tp53*-altered lines developed MPNST and in mice, homozygous inactivation of *Nf1* and *Tp53* in combination with *Suz12* constitute well-established models of MPNST.^34,40,41,42^*TP53* inactivation may also drive chromosomal instability in altered tumors, although the relation of *TP53* mutations to aneuploidy in MPNST remains poorly characterized.^43,44^

## Consensus Recommendations

**Figure 1.**
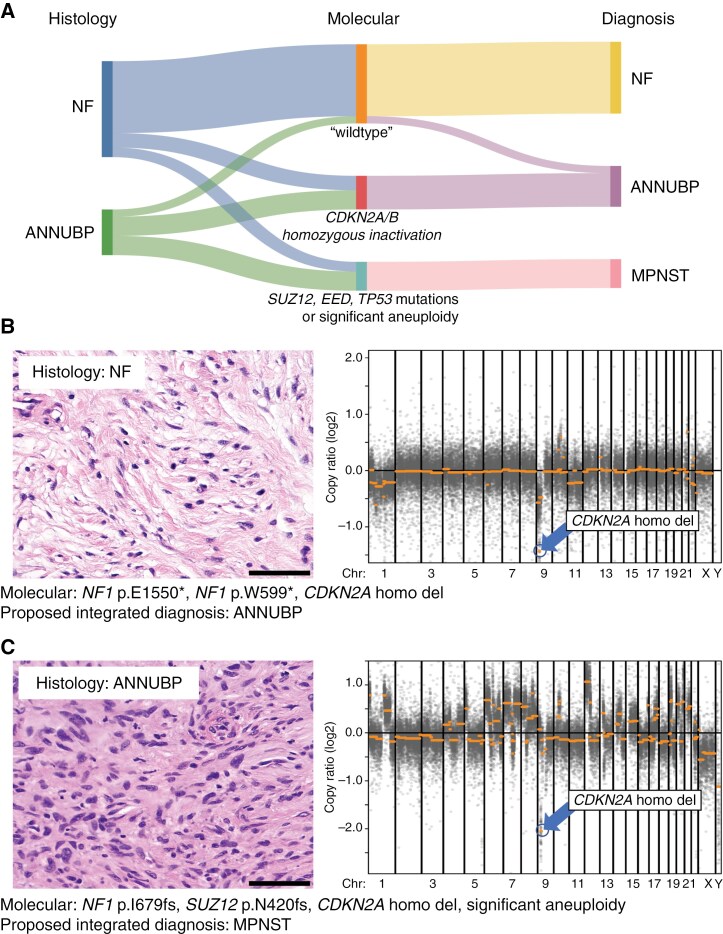
An integrated diagnostic approach for NF1-associated peripheral nerve sheath tumors. (A) Integration of histologic and molecular features would result in reclassification of a subset of tumors with neurofibroma histology but also harboring *CDKN2A/B* homozygous deletion or inactivation as ANNUBP and another subset of tumors with either neurofibroma or ANNUBP histology but also harboring *SUZ12*, *EED*, *TP53* mutations, or significant aneuploidy as MPNST. (B) A case previously diagnosed as a plexiform neurofibroma based on histologic features at the time of resection was found to harbor *CDKN2A/B* homozygous deletion on sequencing and would be reclassified as an ANNUBP. (C) A case previously diagnosed as ANNUBP on core biopsy was found to harbor a clonal *SUZ12* frameshift mutation as well as multiple segmental chromosomal gains and losses and would be reclassified as a MPNST. *CDKN2A/B* homozygous deletion is also noted in this case but is not required for the diagnosis of MPNST. Scale bars, 100 µm. ANNUBP = atypical neurofibromatous neoplasm of uncertain biologic potential; MPNST = malignant peripheral nerve sheath tumors; NF1 = neurofibromatosis type 1.

Given the shift in diagnostic pathology toward the use of integrated histopathologic and genomic approaches, the incorporation of additional molecular strata in the classification of NF1-associated peripheral nerve sheath tumors should be formalized to aid in accurate diagnosis and early identification of malignant transformation to enable appropriate intervention for affected patients.^45^ Here, we provide a consensus-integrated diagnostic approach for ANNUBP and MPNST (Table 2; Figure 1A). As with the prior histologic criteria, the following recommendations are generally not applicable to cutaneous neurofibromas, which almost never transform to MPNST. Based on a review of strong preclinical and clinical evidence, we propose the presence of *CDKN2A/B* biallelic inactivation as a sufficient molecular feature for the diagnosis of ANNUBP, even if the histopathology otherwise qualifies only for neurofibroma (Figure 1B). This would most commonly involve focal gene deletion or inactivating mutation with loss of the wildtype allele. While inactivating mutations involving *CDKN2A/B* are rare in MPNST, they have been associated with similar poor clinical outcomes in other tumor types where *CDKN2A/B* status is incorporated into current grading schemes.^46^ Importantly, we propose heterozygous or subclonal *CDKN2A/B* inactivation (through copy number loss or mutation) in isolation would be insufficient for an integrated diagnosis of ANNUBP. However, heterozygous or subclonal *CDKN2A/B* inactivation in combination with any of the worrisome histologic features would support an ANNUBP diagnosis (Table 2). This could include subclonal focal deletion of *CDKN2A/B* at the chromosome 9p21.3 locus, single copy number loss of chromosome arm 9p, or a subclonal *CDKN2A/B* inactivating mutation. Conversely, lack of *CDKN2A/B* inactivation (either heterozygous or homozygous) in a tumor that otherwise histologically meets the diagnostic criteria for ANNUBP would not alter the diagnosis.

**Table 2.** 2024 Proposed Integrated Consensus Classification Scheme

Diagnosis	Histologic features	Molecular features
NF	Lacks histologic features sufficient for the diagnosis of ANNUBP or MPNST	Lacks molecular features sufficient for the diagnosis of ANNUBP or MPNST
ANNUBP	At least 2 of 4 features with or without *CDKN2A/B* inactivation^a^: (a) cytologic atypia, (b) loss of neurofibroma architecture, (c) hypercellularity, or (d) mitotic index > 1/50 HPFs and <3/10 HPFs	*CDKN2A/B* homozygous inactivation^b^ with or without any ANNUBP histologic features OR *CDKN2A/B* heterozygous inactivation in combination with ≥1 ANNUBP histologic feature (a-d) AND Lacks molecular features sufficient for the diagnosis of MPNST
ANNUBP with increased proliferation	ANNUBP but with mitotic index 3–9/10 HPFs AND Lacks necrosis	Lacks molecular features sufficient for the diagnosis of MPNST
MPNST	At least 10 mf/10 HPFs OR 3–9 mf/10 HPFs combined with necrosis	*SUZ12*/*EED* inactivating mutation, *TP53* inactivating mutation, or significant aneuploidy (segmental gain or loss of at least 8 different chromosome arms)^b^

ANNUBP = atypical neurofibromatous neoplasm of uncertain biologic potential; HPFs = high-power fields; MPNST = malignant peripheral nerve sheath tumors; NF = neurofibroma.

^a^ *CDKN2A/B* homozygous or heterozygous inactivation is not required to define an ANNUBP if 2 of the 4 histologic criteria are present.

^b^Presence of these molecular features is sufficient to make the diagnosis of ANNUBP or MPNST even in the absence of concerning histologic features.

Furthermore, we propose that the presence of either *SUZ12*, *EED*, or *TP53* inactivating mutations or significant aneuploidy serve as sufficient molecular features for a diagnosis of MPNST even in the absence of high-grade histologic features (Figure 1C). While formally defining aneuploidy is context-dependent, we recommend that significant aneuploidy be defined as segmental gain or loss of at least eight different chromosome arms.^31,47–50^ Such molecular features should be used to reclassify lesions, even in the absence of high-grade histologic features. However, we acknowledge the presence of other mechanisms that induce malignant transformation in neurofibromatous peripheral nerve sheath tumors; therefore, these alterations are not essential for the diagnosis of MPNST. Lastly, given the reported clinical and genetic overlap for ANNUBP and low-grade MPNST to date, we propose that “low-grade MPNST” should be renamed as “ANNUBP with increased proliferation.” This recommendation is based on the anecdotal experience of oncologists and surgeons who have observed overly aggressive sarcoma-type therapy for patients with a new diagnosis of low-grade MPNST due to the inclusion of the “malignant” term, when marginal resection may be more appropriate.^8,51^

With the increasing significance of molecular features superseding morphologic features and impacting tumor classification, we also suggest the following at the time of initial diagnostic biopsy to maximize tissue utilization for routine histological, immunohistochemical, and molecular assessment (Table 3).^52^ First, standard equipment should be used for routine image-guided percutaneous core biopsy with 14–18 G biopsy needles. Second, to account for intratumoral heterogeneity, sampling specifically targeting multiple radiologically-concerning areas (eg, increased avidity on fluoro-deoxyglucose positron emission tomography [FDG-PET] or decreased apparent diffusion coefficient (ADC) on diffusion-weighted magnetic resonance imaging), as well as clear labeling of biopsy sites of origin on container labels would help to ensure adequate assessment of the specific regions of interest in these often large and heterogeneous tumors.^19^ Third, as most soft tissue sarcoma sampling protocols call for 4–8 20 mm core biopsies per tumor, we would recommend a minimum of 6 core biopsies be obtained for NF1-associated nerve sheath tumors, as long as it is safe and feasible. Lastly, to minimize tissue depletion during histologic evaluation, the core biopsies should be divided into multiple blocks with no more than 2 core biopsies per block at the time of gross examination. In all cases, careful histologic evaluation, ideally by a subspecialized pathologist, is highly recommended.

**Table 3.** Summary of Considerations for Biopsy Sampling of Peripheral Nerve Sheath Tumors Arising in the Setting of Neurofibromatosis Type 1

Preprocedure imaging considerations
Targeting of radiologically concerning but surgically accessible areas, multiple regions if possible
Sampling considerations
Use 14G to 18 G biopsy needles
Obtain at least 6 core biopsies if feasible
Clearly label separate containers with biopsy site of origin (eg, region #1, FDG-PET avid region)
Tissue processing considerations
No more than 2 cores per formalin-fixed, paraffin-embedded block
Evaluation by subspecialized pathologist

**Figure 2.**
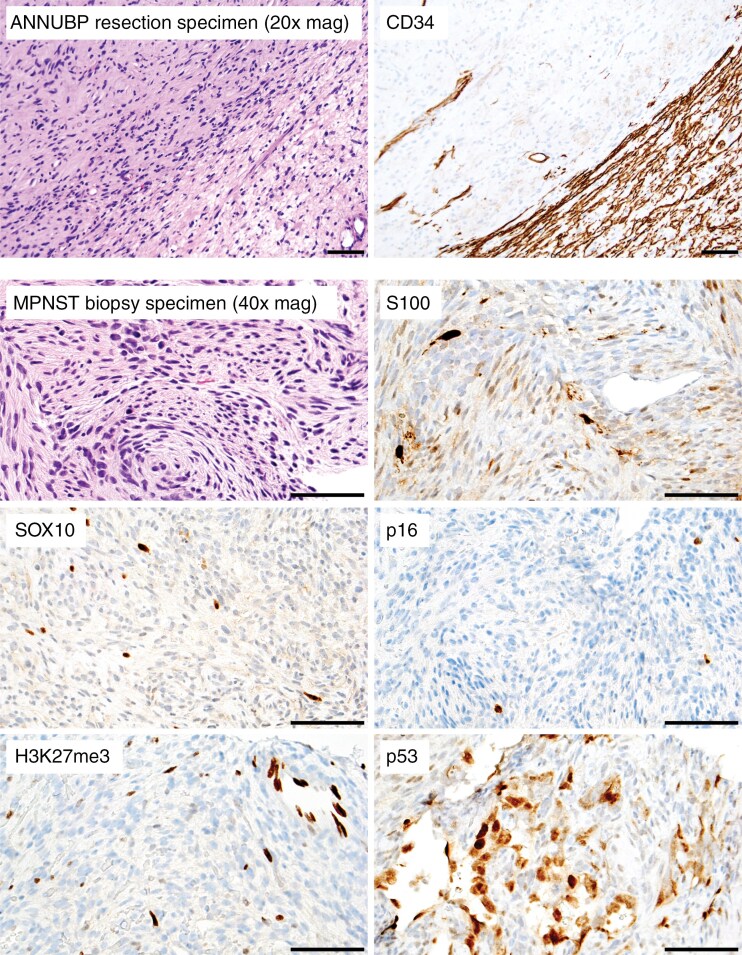
Worrisome immunohistochemical features in NF1-associated peripheral nerve sheath tumors. While difficult to assess on core biopsy specimens, CD34 immunohistochemistry is useful in highlighting the presence of a lattice-like network in a neurofibroma. This network is typically lost in adjacent areas transitioning to ANNUBP and MPNST. Even on core biopsies, decreased expression of S100 and SOX10 is worrisome for a higher-grade lesion, as these markers are typically extensively expressed in neurofibroma. Loss of p16 expression may correlate with underlying *CDKN2A/B* homozygous deletion, and loss of H3K27me3 may correlate with underlying alterations to PRC2 proteins such as SUZ12 or EED. Increased immunoreactivity for p53 may also raise concern. Scale bars, 100 µm. ANNUBP = atypical neurofibromatous neoplasm of uncertain biologic potential; MPNST = malignant peripheral nerve sheath tumors; NF1 = neurofibromatosis type 1; PRC2 = polycomb repressive complex 2.

Subsequent workup should be performed on the block containing the cores with the most worrisome histologic features. The minimum standardized set of histologic features to assess and report for all NF1-associated peripheral nerve sheath tumors would include cytologic atypia, loss of neurofibromatous architecture, hypercellularity, mitotic count per 10 HPF (typically ~2 mm^2^), and necrosis (Table 4). In cases with sufficient tissue, immunohistochemical stains may be performed to clarify the diagnosis and guide block selection for molecular studies. Worrisome immunohistochemical features warranting further molecular assessment include reduced immunoreactivity for SOX10 and/or S100, absence of a CD34-positive lattice-like network, complete loss of p16 expression in tumor cells, complete H3K27me3 loss, increased p53 immunoreactivity (or a null cell pattern), and increased Ki-67 labeling index (Figure 2).^39,53–60^ However, in cases with limited tissue, molecular analysis can be prioritized over immunohistochemistry.

**Table 4.** Example Pathology Reports Incorporating Standardized Set of Histologic Features

NF	Ulnar nerve, mass, and biopsy: plexiform neurofibroma, see comment. Comment: this tumor demonstrates retained neurofibroma architecture without cytologic atypia or hypercellularity. Mitotic figures are inconspicuous.
ANNUBP	Brachial plexus, mass, biopsy: atypical neurofibromatous neoplasm of uncertain biologic potential, see comment. Comment: this tumor demonstrates cytologic atypia, hypercellularity, and focal loss of neurofibroma architecture. Mitotic figures are inconspicuous.
ANNUBP with increased proliferation	Femoral nerve, mass, biopsy: atypical neurofibromatous neoplasm of uncertain biologic potential, see comment. Comment: this tumor does not demonstrate cytologic atypia, hypercellularity, or necrosis. However, focal loss of neurofibroma architecture is noted and the mitotic count reaches 5 mitotic figures per 10 HPFs focally.
MPNST	Sciatic nerve, mass, resection: MPNST, see comment. Comment: This hypercellular tumor is composed of enlarged and atypical tumor cells arranged in fascicles. No well-preserved neurofibroma architecture is noted. Large areas of necrosis are present. The mitotic count reaches 13 mitotic figures per 10 HPFs.

ANNUBP = atypical neurofibromatous neoplasm of uncertain biologic potential; HPFs = high-power fields; MPNST = malignant peripheral nerve sheath tumors; NF = neurofibroma.

Diagnostic molecular studies can be prioritized based on the initial histologic impression. We recommend screening all noncutaneous neurofibromas undergoing diagnostic biopsy to evaluate for molecular features of ANNUBP or MPNST.^61^ The rationale for this recommendation is that referral for biopsy is only made in the presence of worrisome clinical or radiologic features, such as increased avidity on FDG-PET or decreased ADC on diffusion-weighted magnetic resonance imaging. Further, it can be challenging to distinguish benign from transforming nerve sheath tumors based on histologic assessment alone. In cases already meeting histologic criteria for ANNUBP (or ANNUBP with increased proliferation), studies evaluating molecular features of MPNST could either be performed upfront or be reserved for the definitive resection specimen depending on if the information would be used to guide preoperative treatment decisions, such as the extent of resection or neoadjuvant therapy. Seamless communication between the pathologist and the treating clinical team is essential to ensure appropriate use of tissue, and clinical management. In cases already meeting histologic criteria for MPNST, molecular evaluation is not needed for an integrated diagnostic classification but can be performed at the discretion of the multidisciplinary team. As the relevant molecular features include *CDKN2A/B* homozygous or heterozygous inactivation, *SUZ12*, *EED*, or *TP53* inactivating mutations, and significant aneuploidy, assessment with a comprehensive next-generation sequencing panel that includes copy number and zygosity assessment is recommended. As sensitivity for detecting copy number alterations across different assays varies, reported *CDKN2A/B* and aneuploidy results should be interpreted carefully in the context of tumor cellularity and viability. When clinical material is limited, a smaller targeted sequencing panel assessing for *SUZ12*, *EED*, and *TP53* mutations, which are ideally biallelic in nature, along with array comparative genomic hybridization for copy number analysis, would be an alternative approach. While each case should be evaluated in the context of tumor cellularity, we would still consider mutations at a subclonal frequency sufficient for an integrated diagnosis of MPNST.

## Conclusions

Herein, we incorporate recently recognized molecular events into an integrated diagnostic approach for NF1-associated peripheral nerve sheath tumors. We provide a suggested framework for adequate interventional radiology and surgical sampling and recommend molecular profiling for clinically or radiologically worrisome noncutaneous lesions in patients with NF1 to identify diagnostically relevant molecular features, including *CDKN2A/B* inactivation for ANNUBP, as well as *SUZ12*, *EED*, or *TP53* inactivating mutations, or significant aneuploidy for MPNST. The implications of less frequent alterations in *CDKN2A/B* (ie, structural alterations, epigenetic inactivation), as well as other cell cycle and epigenetic regulation genes remain unknown and require further study. We also propose renaming “low-grade MPNST” to “ANNUBP with increased proliferation” to avoid the use of the “malignant” term in this group of tumors with persistent unknown biologic potential. While immunohistochemistry may serve as potential surrogate markers of underlying molecular features (ie, H3K27me3, p16, etc), interrogation with more robust sequencing techniques is recommended given the potential for false positives and false negatives using immunohistochemistry alone.

In the spirit of the prior 2017 consensus conference recommendations, we propose that this refined integrated diagnostic approach for NF1-associated peripheral nerve sheath tumors should continue to evolve in concert with our understanding of these neoplasms.^5,6^ Beyond mutational and copy number assessment, evolving technologies examining DNA methylation and gene expression signatures may further refine classification schemes in the future.^9,52^ Histologic and immunohistochemical assessments are useful for identifying concerning regions of transformation; however, the underlying molecular signatures should further inform diagnostic and risk-stratification schemes, and serve as the framework for therapeutic trials.

## Symposium on Atypical Neurofibroma: State of the Science members

Shivani Ahlawat, Srivandana Akshintala, Kimberly Amrami, Annette Bakker, Allan Belzberg, Jaishri O. Blakeley, Miriam Bredella, Prashant Chittiboina, Wade D. Clapp, Heike Daldrup-Link, Thomas De Raedt, Carina Dehner, Eva Dombi, Garrett Draper, Laura Fayad, Rosalie Ferner, Michael J. Fisher, David H. Gutmann, Andrea M. Gross, Kristina Hawk, Angela Hirbe, Fabian Johnston, Aerang Kim, Bruce R. Korf, David Largaespada, Alexander Lazar, Lu Le, Eric Legius, Adam S. Levin, Calixto-Hope G. Lucas, Ina Ly, Markku Miettinen, David Miller, Carol Morris, Mark Murphey, Luis Parada, Melike Pekmezci, Arie Perry, Christine Pratilas, Martha Quezado, Marcus Ratley, Nancy Ratner, Steven D. Rhodes, Inka Ristow, Fausto Rodriguez, Carlos G. Romo, Eduard Serra Arenas, Steven Sheard, John Shern, Benjamin Siegel, Anat Stemmer-Rachamimov, Taylor R. Sundby, Jeffrey Szymanski, Harish N. Vasudevan, David Viskochil, Brian D. Weiss, Lennart Well, and Brigitte C. Widemann.

## Supplementary material

Supplementary material is available online at *Neuro-Oncology* (https://academic.oup.com/neuro-oncology).

noae235_suppl_Supplementary_Table
